# Variations in Quality of Care by Sex and Social Determinants of Health Among Younger Adults With Acute Myocardial Infarction in the US and Canada

**DOI:** 10.1001/jamanetworkopen.2021.28182

**Published:** 2021-10-20

**Authors:** Valeria Raparelli, Louise Pilote, Brian Dang, Hassan Behlouli, James D. Dziura, Hector Bueno, Gail D’Onofrio, Harlan M. Krumholz, Rachel P. Dreyer

**Affiliations:** 1 Department of Translation Medicine, University of Ferrara, Ferrara, Italy; 2 Faculty of Nursing, University of Alberta, Edmonton, Alberta, Canada; 3 Centre for Outcomes Research and Evaluation, McGill University Health Centre Research Institute, Montreal, Quebec, Canada; 4 Division of Clinical Epidemiology, McGill University Health Centre Research Institute, Montreal, Quebec, Canada; 5 Division of General Internal Medicine, McGill University Health Centre Research Institute, Montreal, Quebec, Canada; 6 Department of Emergency Medicine, Yale University School of Medicine, New Haven, Connecticut; 7 Centro Nactional de Investigaciones Cardiovasculares, Madrid, Spain; 8 Cardiology Department, Hospital Universitario 12 de Octubre, Instituto de Investigacion Sanitaria Hospital 12 de Octubre, Madrid, Spain; 9 Centro de Investigación Biomédica en Red Enfermedades Cardiovasculares, Madrid, Spain; 10 Center for Outcomes Research and Evaluation, Yale–New Haven Health, New Haven, Connecticut; 11 Section of Cardiovascular Medicine, Department of Internal Medicine, Yale School of Medicine, New Haven, Connecticut; 12 Department of Health Policy and Management, Yale School of Public Health, New Haven, Connecticut; 13 Department of Health Policy and Management, Yale School of Public Health, New Haven, Connecticut

## Abstract

**Importance:**

Quality of care of young adults with acute myocardial infarction (AMI) may depend on health care systems in addition to individual-level factors such as biological sex and social determinants of health (SDOH).

**Objective:**

To examine whether the quality of in-hospital and postacute care among young adults with AMI differs between the US and Canada and whether female sex and adverse SDOH are associated with a low quality of care.

**Design, Setting, and Participants:**

This retrospective cohort analysis used data from 2 large cohorts of young adults (aged ≤55 years) receiving in-hospital and outpatient care for AMI at 127 centers in the US and Canada. Data were collected from August 21, 2008, to April 30, 2013, and analyzed from July 12, 2019, to March 10, 2021.

**Exposures:**

Sex, SDOH, and health care system.

**Main Outcomes and Measures:**

Opportunity-based quality-of-care score (QCS), determined by dividing the total number of quality indicators of care received by the total number for which the patient was eligible, with low quality of care defined as the lowest tertile of the QCS.

**Results:**

A total of 4048 adults with AMI (2345 women [57.9%]; median age, 49 [interquartile range, 44-52] years; 3004 [74.2%] in the US) were included in the analysis. Of 3416 patients with in-hospital QCS available, 1061 (31.1%) received a low QCS, including more women compared with men (725 of 2007 [36.1%] vs 336 of 1409 [23.8%]; *P* < .001) and more patients treated in the US vs Canada (962 of 2646 [36.4%] vs 99 of 770 [12.9%]; *P* < .001). Conversely, low quality of post-AMI care (748 of 2938 [25.5%]) was similarly observed for both sexes, with a higher prevalence in the US (678 of 2346 [28.9%] vs 70 of 592 [11.8%]). In adjusted analyses, female sex was not associated with low QCS for in-hospital (odds ratio [OR], 1.05; 95% CI, 0.87-1.28) and post-AMI (OR, 1.07; 95% CI, 0.88-1.30) care. Conversely, being treated in the US was associated with low in-hospital (OR, 2.93; 95% CI, 2.16-3.99) and post-AMI (OR, 2.67; 95% CI, 1.97-3.63) QCS, regardless of sex. Of all SDOH, only employment was associated with higher quality of in-hospital care (OR, 0.72; 95% CI, 0.59-0.88). Finally, only in the US, low quality of in-hospital care was associated with a higher 1-year cardiac readmissions rate (234 of 962 [24.3%]).

**Conclusions and Relevance:**

These findings suggest that beyond sex, health care systems and SDOH that depict social vulnerability are associated with quality of AMI care. Taking into account SDOH among young adults with AMI may improve quality of care and reduce readmissions, especially in the US.

## Key Points

**Question:**

Among young adults with acute myocardial infarction (AMI) in the US and Canada, is quality of care associated with sex, social determinants of health (SDOH), and health care systems?

**Findings:**

In this cohort study of 4048 adults 55 years or younger with AMI, being treated in the US (multipayer system) relative to Canada (single-payer system) was associated with a lower in-hospital and post-AMI quality-of-care score, regardless of all SDOH factors except unemployment. Lower in-hospital quality of care was associated with 1-year readmission rates in the US only.

**Meaning:**

These findings suggest that health care systems and unemployment are associated with quality of AMI care.

## Teaser

This cohort study of young adults aged 55 years or younger with acute myocardial infarction (AMI) in the US and Canada examines whether quality of care for AMI is associated with sex, social determinants of health, and health care systems.

## Introduction

Quality of care and health care gaps for adults 55 years or younger with acute myocardial infarction (AMI) are of immediate concern, because young women experience higher mortality rates than aged-matched men and older women.^1,2,3,4^ To identify factors associated with quality of AMI care, both individual-level characteristics (ie, sex and gendered social determinants of health [SDOH]; ie, the circumstances in which people are born, grow up, live, work, and age, and the systems put in place to offer health care and services to a community)^5^ and health care system resources (ie, structure and financing of systems) should be considered.^6,7^ Universal health care has been promoted to ensure care among vulnerable populations, including young and low-income groups.^8^ In the US, there is a debate about whether to expand or abolish government-based insurance coverage to improve clinical outcomes.^9,10^

Although research has primarily focused on identifying biological mechanisms linked to sex disparities in access to care and adverse outcomes, gendered SDOH also may play a role, as yet understudied in the context of the quality of AMI care in young adults.^11^ Previous studies have shown that feminine personality traits, housework responsibility, and low income were associated with an increased risk of recurrent events and lower access to timely cardiac procedures among young adults with AMI, regardless of sex.^11,12,13^ However, the extent to which quality of AMI care is associated with SDOH and health care systems is unknown.

To address these knowledge gaps, we used data from the 2 largest available prospective cohorts of young adults with AMI: VIRGO (Variation In Recovery: Role of Gender on Outcomes of Young AMI Patients)^14^ and GENESIS-PRAXY (Gender and Sex Determinants of Cardiovascular Disease: From Bench to Beyond-Premature Acute Coronary Syndrome)^15^ studies. Our objectives were to examine whether (1) the quality of in-hospital and postacute care among young adults with AMI differs between the US and Canada; (2) biological sex and SDOH are associated with a low quality of care; and (3) low in-hospital quality of care is associated with cardiac readmissions 1 year after AMI.

## Methods

### Participants and Study Design

Data came from the VIRGO and GENESIS-PRAXY multicenter cohorts^14,15^ designed to investigate factors associated with adverse clinical outcomes in adults 55 years or younger with AMI. The designs of both studies have been described previously.^14,15^ Briefly, a total of 3572 adults with AMI (ratio of women to men, 2:1) in the VIRGO study were recruited across 103 sites in the US and 24 sites in Spain (from August 21, 2008, to May 1, 2012). A total of 1210 adults (392 women) with acute coronary syndrome in the GENESIS-PRAXY study were recruited across 24 sites in Canada, 1 site in the US, and 1 site in Switzerland (from January 1, 2009, to April 30, 2013). For this study, we included only participants with AMI treated in the US (94 of 103 [91.3%] academic hospitals; median bed size, 536) and Canada (18 of 24 [75.0%] academic hospitals; median bed size, 455), for a total of 4048 adults treated at 127 sites. This study followed the Strengthening the Reporting of Observational Studies in Epidemiology (STROBE) reporting guideline. To create a unique data platform, institutional review board approval was obtained at each participating institution, and individuals provided written informed consent for their study participation.

### Data Collection and Measures

In both studies, we collected baseline characteristics and information regarding care received in the 12-month follow-up from medical record abstraction and standardized in-person interviews. Careful harmonization of variables between databases was performed through a recoding process, followed by data quality review and verification from 3 independent researchers (V.R., B.D., and R.P.D.).

#### Sociodemographic and Clinical Characteristics and Gendered SDOH

At baseline, data on age, country, self-reported race, and type of health care system in which the patient was treated (defined by whether coverage was government single payer [Canada] or multipayer [US]) were collected. Race was measured to obtain information on demographic characteristics for descriptive purposes and was analyzed as a potential determinant of the quality of care received. In the US, the health care system is predominantly privately funded through insurance coverage; however, subgroups of individuals are eligible to receive government-funded coverage (ie, Medicaid and Medicare).^10^ In this analysis, we defined the US as a multipayer health care system to account for the combination of different types of insurance.

Prevalence of obesity, hypertension, diabetes, dyslipidemia, current smoking, family history of cardiovascular disease, physical activity, prior AMI, history of renal disease, and alcohol abuse was measured, as well as disease severity, history of depression, and current symptoms of depression (assessed using the *Diagnostic and Statistical Manual of Mental Disorders* [Fourth Edition] criteria).^16^ Self-reported patient sex was abstracted from medical records. Social determinants of health, defined according to the World Health Organization,^5^ that were gendered were collected by self-report at baseline, including (1) identity (level of stress as a personality trait), (2) roles (household primary earner, employment, support for household chores, hours of work per week), (3) relations (marital status and social support), and (4) institutionalized gender (socioeconomic status [SES]; eg, personal income, educational level, job value quality, social standing, policy).^12^

Social support was measured using the ENRICHD (Enhancing Recovery in Coronary Heart Disease) Social Support Instrument.^17,18^ Low social support was defined as a score of 3 or less on at least 2 ENRICHD Social Support Instrument items and a total ENRICHD Social Support Instrument score of 18 or less.^19^ Low SES was defined combining educational attainment (ie, less than high school) and the 2 lowest categories of annual income in both cohorts (ie, ≤US $30 000 and ≤CAD $30 000).

#### Quality of Care Indicators and Cardiac Readmission Outcome

Guided by international recommendations regarding standards of AMI care, quality of care indicators listed in eTable 1 in the Supplement were selected.^20,21,22,23,24,25,26^ We identified quality indicators during in-hospital and post-AMI (1-year post discharge) care.

For each phase of care, we calculated an opportunity-based quality-of-care score (QCS), which was determined by dividing the total number of quality indicators of care received by the total number for which the patient was eligible. Equal weight was given to each indicator as reported previously.^9,27,28^ Each indicator was similarly available in the 2 cohorts. The QCS ranges from 0% to 100%, with higher scores indicating better quality of care.^9,27^ In line with previous research, the opportunity-based composite QCS was split into tertiles.^28^ Low quality of care was defined as the lowest tertile of QCS.

For both data sets, cardiac readmissions were documented using case report files completed by research nurses through telephone interviews and medical record reviews 1 year after hospitalization for the index AMI. To ensure consistency, the major fields of adjudication in the VIRGO study were matched to those collected in the GENESIS-PRAXY study.

### Statistical Analysis

Data were analyzed from July 12, 2019, to March 10, 2021. We compared baseline clinical characteristics, SDOH, and quality of care indicators by sex within and between countries using the unpaired 2-tailed *t* test for continuous variables or the χ^2^ test for categorial variables. Each SDOH was evaluated independently. Multivariable logistic binomial regression models were used to examine the independent effect of sex, SDOH, and health care system on low quality of care as defined by the lowest tertile of QCS. We followed the same process for in-hospital and post-AMI QCS. Potential covariates were selected using a combination of statistical parameters (ie, statistically significant association in the univariate analysis [*P* < .05]) and clinical judgment (eTables 2 and 3 in the Supplement).^1^

A series of 2-way interactions were tested: country-by-sex and individual SDOH-by-country interactions using the model-based Wald test, at 2-tailed α = .05. Analyses were conducted with and without imputation of missing data. The variables with the highest percentage of missingness included post-AMI echocardiogram (22%) and cardiologist visits (33%). The Markov Chain Monte Carlo method was used to impute 5 data sets by filling in missing data of variables included in the regression models. The imputation model was calculated with all potential confounders with the lowest tertile QCS as the dependent variable. All analyses were performed in the 5 imputed data sets, and results of individual data sets were combined with consideration for the variation between results of the 5 data sets.

To account for within-site clustering and random site-to-site variability, we applied a generalized linear mixed-modeling framework. All statistical analyses were conducted using SAS, version 9.4 (SAS Institute Inc), with 2-tailed tests for statistical significance indicated by α = .05.

## Results

### Baseline Characteristics

Overall, of the 4048 individuals enrolled, 3004 (74.2%) were from the US. The median age was 49 (IQR, 44-52) years; 2345 individuals (57.9%) were women and 1703 (42.1%) were men; 906 (22.4%) were Black and 3142 (77.6%) were White. Women, especially in the US, exhibited a more adverse cardiac risk factor profile (Table 1). In addition, in both countries, women had a greater burden of detrimental SDOH, such as low SES (1006 of 2345 [42.9%] vs 409 of 1703 [24.0%]), unemployment (994 of 2345 [42.4%] vs 414 of 1703 [24.3%]), living alone/without a partner (1293 of 2345 [55.1%] vs 618 of 1703 [36.3%]), high stress (1272 of 2297 [55.4%] vs 666 of 1659 [40.1%]), and less support for household chores (1420 of 2345 [60.6%] vs 1141 of 1703 [67.0%]) than men.

**Table 1.** Baseline Demographics, Clinical Characteristics, and Gendered Social Factors Stratified by Sex and Country

	Canada (n = 1044)		US (n = 3004)		*P* value for Canada vs US	
	Women (n = 331)	Men (n = 713)	Women (n = 2014)	Men (n = 990)	Women	Men
Age, mean (SD), y	48.5 (5.7)	47.8 (6.0)	47.2 (6.3)	47.1 (5.9)	<.001	.01
Race					<.001	.007
Black	55 (16.6)	159 (22.3)	523 (26.0)	169 (17.1)		
White	276 (83.4)	554 (77.7)^a^	1491 (74.0)	821 (82.9)^b^		
Cardiac risk factors						
Obesity	137 (41.4)	277 (38.8)	1113 (55.3)	474 (47.9)^b^	<.001	<.001
Hypertension	179 (54.1)	314 (44.0)^a^	1351 (67.1)	637 (64.3)	<.001	<.001
Diabetes	72 (21.8)	100 (14.0)^a^	802 (39.8)	262 (26.5)^b^	<.001	<.001
Dyslipidemia	171 (51.7)	392 (55.0)	1683 (83.6)	915 (92.4)^b^	<.001	<.001
Current smoking	142 (42.9)	261 (36.6)	608 (30.2)	299 (30.2)	<.001	.006
Family history of CVD^c^	57 (21.1)	92 (16.7)	1350 (67.3)	659 (67.0)	<.001	<.001
Physically inactive^c^	66 (19.9)	141 (19.8)	754 (37.4)	309 (31.2)^a^	<.001	<.001
Comorbidities/medical history						
Prior AMI	53 (16.0)	89 (12.5)	413 (20.5)	228 (23.0)	.06	<.001
History of renal disease^c^	17 (5.1)	33 (4.6)	255 (12.7)	84 (8.5)^b^	<.001	.002
Alcohol abuse	109 (32.9)	250 (35.1)	557 (27.7)	460 (46.5)^b^	.049	<.001
History of depression	85 (25.7)	143 (20.1)^a^	979 (48.6)	242 (24.4)^b^	<.001	.03
Symptoms of depression (*DSM-IV*)^c^	71 (36)	88 (24.7)^a^	414 (20.6)	109 (11.1)^b^	<.001	<.001
Disease severity						
AMI type						
STEMI	180 (54.4)	485 (68.0)^b^	924 (45.9)	569 (57.5)^b^	.004	<.001
NSTEMI	151 (45.6)	228 (32.0)^b^	1090 (54.1)	421 (42.5)^b^		
SDOH						
Low SES	67 (20.2)	96 (13.5)^a^	959 (47.6)	313 (31.6)^b^	<.001	<.001
Current employment	220 (66.5)	576 (80.8)^b^	1131 (56.2)	713 (72.0)^b^	<.001	<.001
Time at work, mean (SD), h/wk	38.5 (12.5)	46.8 (12.8)^b^	38.9 (13.0)	46.2 (13.6)^b^	.73	.44
Married or living with partner	196 (59.2)	470 (65.9)^a^	1056 (52.4)	615 (62.1)^b^	.02	.11
Primary earner^c^	94 (35.9)	412 (74.5)^b^	1488 (73.9)	740 (75.0)	<.001	.81
High burden of stress^c^	179 (58.3)	291 (42.8)^b^	1093 (54.9)	375 (38.3)^b^	.27	.07
Support for household chores	162 (48.9)	474 (66.5)^b^	1258 (62.5)	667 (67.4)^a^	<.001	.15
Low social support	87 (26.3)	149 (20.9)^a^	414 (20.6)	212 (21.4)	<.001	.58

Abbreviations: AMI, myocardial infarction; CVD, cardiovascular disease; *DSM-IV*, *Diagnostic and Statistical Manual of Mental Disorders* (Fourth Edition); NSTEMI, non–ST-segment elevation myocardial infarction; SDOH, social determinants of health; SES, socioeconomic status; STEMI, ST-segment elevation myocardial infarction.

^a^ *P* < .05 for comparison between women and men.

^b^ *P* < .001 for comparison between women and men.

^c^ Missing data of 10% or less.

### In-Hospital Quality of Care and Quality Indicators

The in-hospital QCS was divided into tertiles: low (≤66%; 1061 [31.1%]), intermediate (67%-76%; 875 [25.6%]), and high (≥77%; 1480 [43.3%]). The low QCS was more prevalent in the US than in Canada (962 of 2646 [36.4%] vs 99 of 770 [12.9%]; *P* < .001). This difference was mainly driven by lower cardiac rehabilitation counseling (1373 of 3004 [45.7%] vs 809 of 1044 [77.5%]), the lower proportion of patients with non–ST-segment elevation myocardial infarction (NSTEMI) at high risk undergoing reperfusion strategy (1100 of 1511 [72.8%] vs 240 of 379 [63.3%]), and the less likely discharge prescription of antiplatelets (1981 of 3004 [65.9%] vs 871 of 1044 [83.4%]). The low QCS was more prevalent in women than in men (725 of 2007 [36.1%] vs 336 of 1049 [23.8%]; *P* < .001). Women had a lower unadjusted in-hospital QCS than men, lower in the US (median, 75 [IQR, 62-85] vs 75 [62-87] in men) compared with Canada (median, 77 [IQR, 66-88] vs 78 [IQR, 75-89] in men) (Table 2). Compared with men, women with STEMI in both countries were more likely to exceed the benchmarks for door-to-balloon time and door-to-needle time (Canadian, 66 of 180 [36.7%] vs 184 of 924 [19.9%]; US, 317 of 924 [34.3%] vs 180 of 569 [31.6%]). The proportion of patients with NSTEMI undergoing reperfusion was lower in Canada (240 of 379 [63.3%]) than in the US (1100 of 1511 [72.8%]). In addition, less than 50% of US participants received cardiac rehabilitation counseling on discharge, and women were less likely to be counseled than men, more so in Canada (243 of 331 [73.4%] vs 566 of 713 [79.4%]) than in the US (902 of 2019 [44.7%] vs 471 of 990 [47.6%]). In both countries, secondary prevention therapy was less likely to be prescribed at discharge in women compared with men, more so in the US. Male sex (OR, 0.55; 95% CI, 0.47-0.64), low SES (OR, 1.38; 95% CI, 1.19-1.61), unemployment (OR, 0.59; 95% CI, 0.51-0.69), and various comorbidities (eg, OR for diabetes, 1.42; 95% CI, 1.22-1.66) were associated with the low in-hospital QCS (eTable 2 in the Supplement).

**Table 2.** In-Hospital and Post-AMI Quality Indicators of Care Stratified by Sex and Country

Quality indicator	Canada (n = 1044)		US (n = 3004)		*P* value for Canada vs US	
	Women (n = 331)	Men (n = 713)	Women (n = 2014)	Men (n = 990)	Women	Men
No. of patients with in-hospital care	225	545	1782	864	NA	NA
In-hospital quality indicators						
In-hospital QCS, median (IQR)	77 (66-88)	78 (75-89)^a^	75 (62-85)	75 (62-87)^a^	<.001	<.001
In-hospital QCS, No. (%)						
Low tertile (≤66%)	40 (17.8)	59 (10.8)	685 (38.4)	277 (32.1)^b^	<.001	<.001
Intermediate tertile (67%-76%)	57 (25.3)	135 (24.8)	470 (26.4)	213 (24.7)^b^		
High tertile (≥77%)	128 (56.9)	351 (64.4)	627 (35.2)	374 (43.3)^b^		
Patients with STEMI^c^						
Any reperfusion therapy^d^	146 (91.3)	418 (92.1)	783 (85.5)	487 (86.5)	.049	.005
Door-to-balloon time exceed benchmark	66 (61.7)	184 (57.9)	317 (47.10)	180 (42.3)	.005	<.001
Door-to-needle time exceed benchmark	24 (64.9)	51 (47.7)	42 (61.8)	21 (37.5)^b^	.75	.21
Patients with NSTEMI^e^						
Any reperfusion therapy received^d^	85 (90.4)	155 (95.1)	799 (77.7)	301 (74.5)	.004	<.001
All patients with AMI, No. (%)						
Stress test in conservatively treated individuals^f^	1 (5.0)	6 (16.7)	3 (4.5)	0	.43	.048
Echocardiogram before discharge^d^	204 (61.6)	450 (63.1)	1385 (69.0)	672 (67.9)	.008	.033
Recommended counseling	121 (36.6)	231 (32.4)	632 (31.4)	321 (32.4)	.06	.99
Cardiac rehabilitation counseling	243 (73.4)	566 (79.4)^b^	902 (44.8)	471 (47.6)	<.001	<.001
Smoking counseling	165 (49.8)	330 (46.3)	1331 (66.1)	658 (66.5)	<.001	<.001
Diet counseling	236 (71.3)	510 (71.5)	1839 (91.3)	915 (92.4)	<.001	<.001
Aspirin at discharge	315 (95.2)	698 (97.9)^b^	1864 (92.6)	939 (94.8)^b^	.09	.001
P2Y12 receptor antagonist at discharge	253 (76.4)	626 (87.8)^a^	1359 (67.5)	710 (71.7)^b^	.001	<.001
DAPT at discharge	251 (75.8)	620 (87.0)^a^	1293 (64.2)	688 (69.5)^b^	<.001	<.001
Statins at discharge	298 (90.0)	675 (94.7)^b^	1819 (90.3)	940 (94.9)^a^	.87	.79
β-Blockers at discharge	275 (83.1)	620 (87.0)	1803 (89.5)	931 (94.0)^a^	<.001	<.001
No. with post-AMI care	194	398	1593	753	NA	NA
Post-AMI quality indicators						
Post-AMI QCS, median (IQR)	50 (50-75)	75 (50-75)^a^	50 (25-75)	50 (25-75)	<.001	<.001
Post-AMI QCS, No. (%)						
Low tertile (≤25%)	31 (16.0)	39 (9.8)^b^	469 (29.4)	209 (27.8)	<.001	<.001
Intermediate tertile (26%-74%)	71 (36.6)	136 (34.2)^b^	609 (38.2)	319 (42.4)		
High tertile (≥75%)	92 (47.4)	223 (56.0)^b^	515 (32.3)	225 (29.9)		
Primary health care clinician visits, median (IQR)	3 (1-6)	3 (1-5)	2 (1-4)	2 (1-4)	.08	.006
No.of cardiologist visits, median (IQR)	2 (1-3)	2 (1-3)	3 (2-4)	2 (2-4)	<.001	<.001
Echocardiogram performed, No. (%)^d^	85 (31.72)	170 (30.6)	360 (22.6)	152 (20.1)	.001	<.001
Long-term statin therapy, No. (%)^d^	164 (78.1)	389 (87.4)^b^	700 (34.8)	316 (32.1)	<.001	<.001
DAPT at 12 mo, No. (%)^d^	81 (38.6)	199 (44.7)	753 (37.4)	394 (40.0)	.74	.10

Abbreviations: AMI, acute myocardial infarction; DAPT, dual antiplatelet therapy; NA, not applicable; NSTEMI, non–ST-segment elevation myocardial infarction; QCS, quality-of-care score; STEMI, ST-segment elevation myocardial infarction.

^a^ *P* < .001 for comparison between women and men.

^b^ *P* < .05 for comparison between women and men.

^c^ Canada, n = 665 (180 women, 485 men); US, n = 1493 (924 women, 569 men).

^d^ Missing data of at least 10%.

^e^ Canada, n = 56 (20 women, 36 men); US, n = 89 (67 women, 22 men).

^f^ Canada, n = 379 (151 women, 228 men); US, n = 1511 (1090 women, 421 men).

### Factors Associated With Low In-Hospital Quality of Care

**Figure 1.**
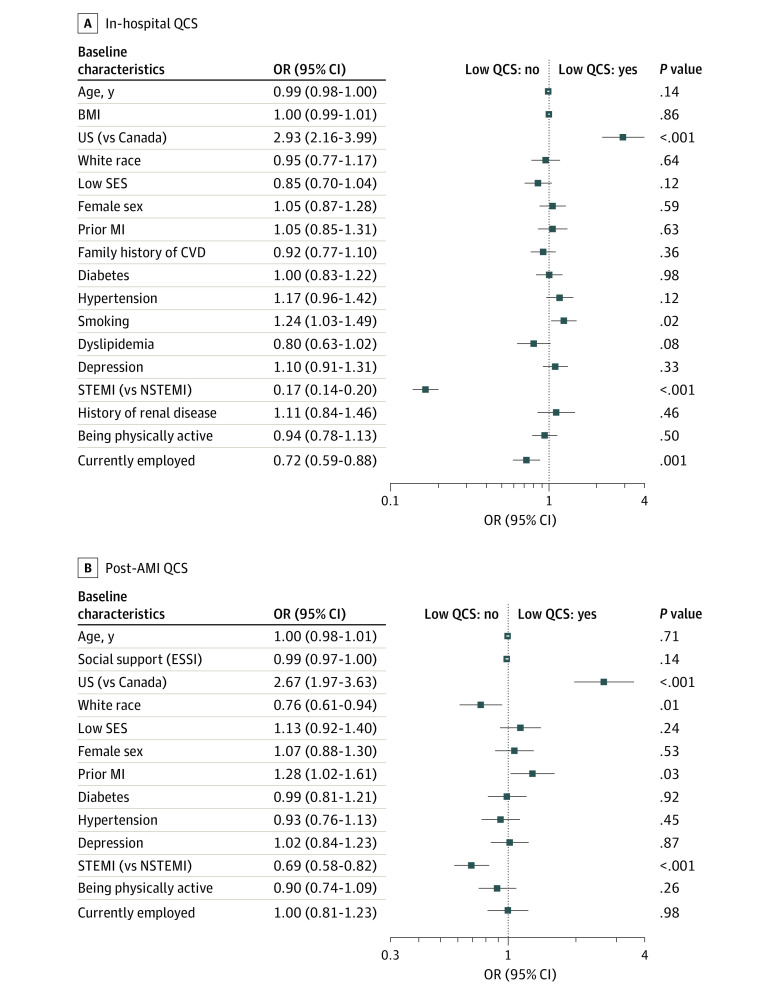
Forest Plots Illustrating the Effect of Adjustment on the Associations Between Sex, Social Determinants of Health, and Health Care System With Low Quality-of-Care Score (QCS) Health care system is compared between single payer (Canada) and multipayer (US). A, Covariates included in the model for lower in-hospital QCS include age, sex, body mass index (BMI), country, self-reported race, low socioeconomic status (SES), prior myocardial infarction (MI), family history of cardiovascular disease (CVD), diabetes, hypertension, smoking, dyslipidemia, history of depression, type of acute MI (AMI), history of renal disease, physical activity, and employment. B, Covariates included in the model for lower post-AMI QCS include age, sex, country, self-reported race, ENRICHD (Enhancing Recovery in Coronary Heart Disease) Social Support Instrument (ESSI) score, low SES, prior MI, diabetes, hypertension, history of depression, type of AMI, physical activity, and employment.

In the multivariable model (Figure 1A), factors that were associated with low QCS included being treated in the US (OR, 2.93; 95% CI, 2.16-3.99) and smoking (OR, 1.24; 95% CI, 1.03-1.49). Conversely, being employed (OR, 0.72; 95% CI, 0.59-0.88) and experiencing a STEMI (OR, 0.17; 95% CI, 0.14-0.20) were inversely associated with low QCS. Similar findings were obtained when the multivariable model was performed with imputations (eTable 3 in the Supplement) and accounting for the potential effect of within-site clustering (eTable 4 in the Supplement). None of the 2-way interactions among sex, SDOH, country, and low in-hospital QCS were significant (eTable 5 in the Supplement).

### Post-AMI Quality of Care and Quality Indicators

The post-AMI QCS was grouped into low (≤25%; 748 [25.5%), intermediate (26%-74%; 1135 [38.6%]), and high (≥75%; 1055 [35.9%]) tertiles. The post-AMI QCS was equally low in both sexes; however, participants fared worse in the US compared with Canada (678 of 2346 [28.9%] vs 70 of 592 [11.8%]) (Table 2). This between-country difference was mainly driven by fewer medical visits after discharge, less reassessment of ventricular function by echocardiogram, and lower adherence to statin therapy. In the US, women received a median of 3 (IQR, 2-4) post-AMI follow-up cardiologist visits compared with 2 (IQR, 2-4) for men, with a low proportion receiving echocardiogram reassessments (360 of 1593 women [22.6%] and 152 of 756 men [20.1%]) and adherence to statins (700 of 2013 women [34.8%] and 316 of 984 men [32.1%]) or antiplatelets (753 of 2013 women [37.4%] and 394 of 985 men [40.0%]). Conversely in Canada, both sexes received similar primary care clinician follow-up visits (median, 3 [IQR for women, 1-6; IQR for men, 1-5]) and echocardiogram reassessments (85 of 268 women [31.7%] and 170 of 556 men [30.6%]), but women were less likely to use statins than men (164 of 210 women [78.1%] vs 389 of 495 men [78.6%]) (Table 2). Clinical characteristics (eg, OR for prior MI, 1.49; 95% CI, 1.21-1.83) and SDOH, including social support (ESSI score) (OR, 0.98; 95% CI, 0.97-0.99), household support (OR, 0.83; 95% CI, 0.69-0.98), and being employed (OR, 0.75; 95% C1, 0.63-0.89), were significantly associated with a low post-AMI QCS (eTable 6 in the Supplement).

### Factors Associated With Low Post-AMI Quality of Care

In the multivariable model (Figure 1B), the factors associated with the low post-AMI QCS included being treated in the US (relative to Canada) (OR, 2.67; 95% CI, 1.97-3.63) and having a prior MI (OR, 1.28; 95% CI, 1.02-1.61), whereas White race (OR, 0.76; 95% CI, 0.61-0.94) and experiencing a STEMI (OR, 0.69; 95% CI, 0.58-0.82) were inversely associated with the low QCS. Neither female sex (OR, 1.07; 95% CI, 0.88-1.30) nor SDOH (OR for social support [ESSI score], 0.99 [95% CI, 0.97-1.00]; OR for low SES, 1.13 [95% CI, 0.92-1.40]; OR for currently employed, 1.00 [95% CI, 0.81-1.23]) were independently associated with low QCS. Similar findings were obtained when the multivariable model was performed with imputation (eTable 7 in the Supplement) and accounting for the potential effect of within-site clustering (eTable 8 in the Supplement). None of the 2-way interactions among sex, SDOH, country, and low post-AMI QCS were significant (eTable 9 in the Supplement).

### Association Between Cardiac Readmission and Low In-Hospital QCS

**Figure 2.**
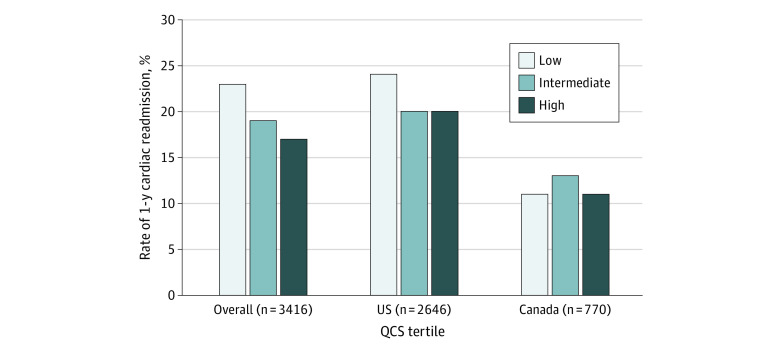
Rates of Cardiac Readmission at 1 Year Stratified by Tertiles of In-Hospital Quality-of-Care Score (QCS) in the Overall Cohort and in Each Country Low QCS indicates 66% or less; intermediate, 67% to 76%; and high, 77% or greater. For overall cohort, cardiac readmissions were 245 of 1061 patients with low QCS (23.1%), 163 of 875 patients with intermediate QCS (18.6%), and 250 of 1480 patients with high QCS (16.9%) (*P* = .007). In the US, cardiac readmissions were 234 of 962 patients with low QCS (24.3%), 139 of 683 patients with intermediate QCS (20.3%), and 200 of 1001 patients with high QCS (19.9%) (*P* = .04). In Canada, cardiac readmissions were 11 of 99 patients with low QCS (11.1%), 24 of 192 patients with intermediate QCS (12.5%), and 53 of 479 patients with high QCS (11.1%) (*P* = .86).

From the original cohort (n = 4048), 3416 young adults with in-hospital QCS had available 1-year follow-up data for readmission. The rate of cardiac readmission was 19.4% (n = 661). Cardiac readmissions were more prevalent among individuals in the low QCS (245 of 1061 [23.1%]) than in those with intermediate (163 of 875 [18.6%]) or high QCS (250 of 1480 [16.9%]; *P* = .007) (Figure 2). However, when stratifying by country, the association between the low in-hospital QCS and cardiac readmissions persisted only in the US (234 of 962 [24.3%]) (Figure 2).

## Discussion

In this study, low quality of care for AMI in young adults was prevalent and was higher among women during the in-hospital phase. These differences were more marked in the US than in Canada. Of note, low quality of in-hospital care was associated with an increased risk of cardiac readmission in the US relative to Canada. Being employed was associated with higher in-hospital quality of care. These findings suggest that the type of health care system and SDOH are associated with quality of care in young adults with AMI, regardless of sex. Women with AMI exhibited a more adverse cardiac risk factor profile and were more socially vulnerable than men, more markedly in the US. Unemployment was the SDOH most importantly associated with the lowest quality of care. Thus, a country with single-payer systems (eg, Canada) and smaller inequalities in access to health services based on SDOH (potentially because of universal coverage) appeared to attenuate inequities in access to high quality of care.^7^ In particular, women are more likely to be unemployed and have a low SES; hence, they are also more likely to experience gaps in health insurance coverage, which could affect the quality of care.^29^ Furthermore, an association between the lowest quality of care received and rate of 1-year cardiac readmission was evident only in US, suggesting that employment and likely insurance status affect readmissions after AMI through a lower quality of care.

Regarding the in-hospital quality of AMI care, although low cardiac rehabilitation referral rates affected women and men equally in the US, women experienced a lower referral rate in Canada. In addition, our findings extend prior observations of underutilization of secondary prevention in the US among women with AMI,^30^ likely related to an underestimation of their risk or less access to prescription drugs.^31^ Finally, the postdischarge period appears to be the most vulnerable window for young adults with AMI, because the QCS was largely suboptimal in both sexes. Cardiovascular secondary prevention was largely inadequate, particularly for statin and antiplatelet therapy, in accordance with prior literature^32,33^ showing poor cardiovascular medication adherence after AMI, with related increased rate of adverse outcomes.

This study extends the prior literature in several ways. First, we have provided a comprehensive overview of how different health care systems modify the effect of sex and gendered SDOH on quality of AMI care in young adults in North America. The only prior study^27^ focused solely on in-hospital quality of care among elderly from single-payer health care systems (in the UK and Israel) demonstrated that women had higher rates of mortality and were less likely to receive guideline-indicated care than men. Second, no prior work captured a wealth of gendered SDOH for a better understanding of sex differences in AMI outcomes.^29^ Finally, we analyzed the 2 largest prospective studies of young adults with AMI to date, an understudied yet growing population; these studies collected unique sex-related data to estimate their effect on quality of care.

Our findings have significant public health implications. The US health care system should address the in-hospital AMI care of young adults who exhibit an adverse clustering of clinical characteristics and SDOH pending the expansion of government-funded care. The strong association between the lowest QCS and cardiac readmissions poses an urgent issue to tackle in the context of the US health care system costs, aside from post-AMI well-being. These recommendations inform the US debate about allocating additional funding for the Affordable Care Act or abolishing it altogether.^9,34^ Finally, the fact that the post-AMI QCS was poor in both Canada and the US suggests that increased efforts should be geared toward promoting cardiovascular secondary prevention in young adults with AMI. The importance of gendered SDOH has been at the forefront of the COVID-19 pandemic, and attention to these factors should span all aspects of health care.

### Limitations

Our findings should be interpreted in light of potential limitations. First, in determining in-hospital QCS, we could not account for some indicators unavailable in the merged data set (eg, left ventricular systolic dysfunction), and we weighted the indicators the same even if some may be more clearly associated with AMI outcomes. The difference between Canada and the US in reperfusion for NSTEMI might be related to the missing information on the pathogenetic mechanism for AMI (type 1 vs non–type 1). Moreover, the post-AMI QCS has not been computed previously; however, we applied the same statistical approach as the in-hospital QCS^27^ based on international guidelines.^20,21,22,23,24,25,26^ Second, some gendered factors were not captured, such as caregiver roles, which could also be associated with quality of care.^13^ Third, we could only provide a comparison between health care systems with or without universal coverage; therefore, we cannot confirm the possibility that type of insurance coverage in the multipayer US system directly affected quality of care. However, the fact that employment was associated with QCS only in the US suggests that insurance level plays an important role in quality of care, particularly because employers offer commercial insurance benefits that often exceed those offered by government subsidies. Furthermore, the differences in quality of care between countries could have been related to the mix of academic vs nonacademic centers. Although quality of care appeared lower in the US, a greater proportion of centers were academic, perhaps reflecting a different patient mix in terms of health insurance. Fourth, although White race was independently associated with access to care, we recognize that the diversity in racial groups between the US and Canada precludes comparisons. Fifth, contraindications to specific quality measures included in the QCS might have affected the QCS; for example, the reasons why medications were not prescribed were not available in the merged database. Last, we used a broad definition of SDOH^5^ based on individual level data, but we could not account for neighborhood-level characteristics that might intersect with individual variables in their association with quality of AMI care.

## Conclusions

In this cohort study, the health care system in which a patient is treated and SDOH that depict a higher social vulnerability were independently associated with quality of AMI care. Low in-hospital and post-AMI care was predominant in young adults treated in the US compared with Canada regardless of sex. Unemployment was the main SDOH associated with lower in-hospital quality of care. Notably, low in-hospital quality of care was associated with higher readmission rates in US only, suggesting a heightened focus on SDOH to improve access to high-quality care.

## Notes

Supplement. **eTable 1.** Quality Indicators for Each Phase of AMI Care **eTable 2.** Univariate Association Among Sex, Social Determinants of Health, and the Lowest In-Hospital QCS Tertile **eTable 3.** Multivariable Model With Imputed Values for the Lowest In-Hospital QCS Tertile **eTable 4.** Multivariable Model for the Lowest In-Hospital QCS Tertile Using Random Effect for Within-Hospital Clustering **eTable 5.** Two-Way Country-by-Sex and Individual SDOH-by-Country Interactions for the Lowest In-Hospital QCS Tertile **eTable 6.** Univariate Association Among Sex, Social Determinants of Health, and the Lowest Post-AMI QCS Tertile **eTable 7.** Multivariable Model With Imputed Values for the Lowest Post-AMI QCS Tertile **eTable 8.** Multivariable Model for the Lowest Post-AMI QCS Tertile Using Random Effect for Within-Hospital Clustering **eTable 9.** Two-Way Country-by-Sex and Individual SDOH-by-Country Interactions for the Lowest In-Hospital QCS Tertile **eTable 10.** Data Dictionary and Summary of the Harmonized Variables
eReferences
