# Supplementary material for: Variations in Quality of Care by Sex and Social Determinants of Health Among Younger Adults With Acute Myocardial Infarction in the US and Canada
Source: JAMA Netw Open. 2021 Oct 20;4(10):e2128182. doi: 10.1001/jamanetworkopen.2021.28182 (PMC8529414; doi:10.1001/jamanetworkopen.2021.28182)
Supplement: Supplement. — eTable 1. Quality Indicators for Each Phase of AMI Care eTable 2. Univariate Association Among Sex, Social Determinants of Health, and the Lowest In-Hospital QCS Tertile eTable 3. Multivariable Model With Imputed Values for the Lowest In-Hospital QCS Tertile eTable 4. Multivariable Model for the Lowest In-Hospital QCS Tertile Using Random Effect for Within-Hospital Clustering eTable 5. Two-Way Country-by-Sex and Individual SDOH-by-Country Interactions for the Lowest In-Hospital QCS Tertile eTable 6. Univariate Association Among Sex, Social Determinants of Health, and the Lowest Post-AMI QCS Tertile eTable 7. Multivariable Model With Imputed Values for the Lowest Post-AMI QCS Tertile eTable 8. Multivariable Model for the Lowest Post-AMI QCS Tertile Using Random Effect for Within-Hospital Clustering eTable 9. Two-Way Country-by-Sex and Individual SDOH-by-Country Interactions for the Lowest In-Hospital QCS Tertile eTable 10. Data Dictionary and Summary of the Harmonized Variables eReferences [file jamanetwopen-e2128182-s001.pdf]

Supplementary Online Content

Raparelli V, Pilote L, Dang B, et al. Variations in quality of care by sex and social determinants of health among younger adults with acute myocardial infarction in the US and Canada. *JAMA Netw Open*. 2021;4(10):e2128182. doi:10.1001/jamanetworkopen.2021.28182

eTable 1. Quality Indicators for Each Phase of AMI Care

eTable 2. Univariate Association Among Sex, Social Determinants of Health, and the Lowest In-Hospital QCS Tertile

eTable 3. Multivariable Model With Imputed Values for the Lowest In-Hospital QCS Tertile

eTable 4. Multivariable Model for the Lowest In-Hospital QCS Tertile Using Random Effect for Within-Hospital Clustering

eTable 5. Two-Way Country-by-Sex and Individual SDOH-by-Country Interactions for the Lowest In-Hospital QCS Tertile

eTable 6. Univariate Association Among Sex, Social Determinants of Health, and the Lowest Post-AMI QCS Tertile

eTable 7. Multivariable Model With Imputed Values for the Lowest Post-AMI QCS Tertile

eTable 8. Multivariable Model for the Lowest Post-AMI QCS Tertile Using Random Effect for Within-Hospital Clustering

eTable 9. Two-Way Country-by-Sex and Individual SDOH-by-Country Interactions for the Lowest In-Hospital QCS Tertile

eTable 10. Data Dictionary and Summary of the Harmonized Variables

eReferences.

This supplementary material has been provided by the authors to give readers additional information about their work.

© 2021 Raparelli V et al. *JAMA Network Open*.

eTable 1. Quality Indicators for Each Phase of AMI Care

In hospital STEMI care			In-Hospital NSTEMI Care		
**Quality Indicators**	**Definition**	**References**	**Quality Indicators**	**Definition**	**References**
1. Aspirin at discharge	Patients with AMI who are prescribed aspirin at hospital discharge	1-2	1. Aspirin at discharge	Patients with AMI who are prescribed aspirin at hospital discharge	1-2
2. Beta-blockers at discharge	Patients with AMI who are prescribed a beta blocker at hospital discharge	1-2	2. Beta-blockers at discharge	Patients with AMI who are prescribed a beta blocker at hospital discharge	1-2
3. Statins at discharge	Patients with AMI who are prescribed a high-intensity statin at hospital discharge	1-3	3. Statins at discharge	Patients with AMI who are prescribed a high-intensity statin at hospital discharge	1-3
4. Echocardiogram (assessment of ventricular function)	Patients with AMI with documentation in the hospital record that LVEF assessment, which can be either qualitative or quantitative, is done during the hospitalization	1-3	4. Echocardiogram (assessment of ventricular function)	Patients with AMI with documentation in the hospital record that LVEF assessment, which can be either qualitative or quantitative, is done during the hospitalization	1-3
5a. Time to fibrinolytic therapy (<=30 minutes)	Patients with acute STEMI, or its equivalent, who receive fibrinolytic therapy (as the primary reperfusion modality) with a time from hospital arrival to fibrinolysis ≤30 min	1-2	5. Stress test in conservatively treated patients (i.e. patients have not receiving invasive angiography)	Patients with AMI, who are initially conservatively managed (have not received invasive coronary angiography)	2-3

© 2021 Raparelli V et al. *JAMA Network Open*.

5b. Time to primary percutaneous coronary intervention (<=90 minutes)	Patients with acute STEMI, or its equivalent, who receive primary PCI during the hospital stay with a time from first medical contact-to-device time ≤90 min.	1-2	6. Invasive angiography performed	Patients with acute NSTEMI, who are at high or intermediate risk and who receive an invasive strategy within 24 or 72 h	2-3
5c. Stress test in conservatively treated patients (i.e. patients have not receiving invasive angiography)	Patients with AMI, who are initially conservatively managed (have not received invasive coronary angiography)	2	7. Cardiac rehabilitation referral	patients hospitalized with AMI who are referred to an outpatient cardiac rehabilitation	1-2
6. Any Reperfusion Therapy Received	Patients with acute STEMI, or its equivalent, who receive fibrinolytic therapy or primary PCI.	1-2	8. P2Y12 receptor inhibitor prescribed at discharge	Patients with AMI who are prescribed an appropriate P2Y12 receptor inhibitor at hospital discharge	2
7. Cardiac rehabilitation referral	Patients hospitalized with AMI who are referred to an outpatient cardiac rehabilitation	1-2	9. Recommended discharge counseling (smoking cessation advice/counselling + cardiac rehabilitation + diet)	Patients who receive counseling regarding smoking cessation, referral to a cardiac rehabilitation program, and are advised on appropriate diet for secondary prevention prior to discharge.	4-5
8. P2Y12 receptor inhibitor at discharge	Patients with AMI who are prescribed an appropriate P2Y12 receptor inhibitor at hospital discharge	2			

© 2021 Raparelli V et al. *JAMA Network Open*.

9. Recommended discharge counseling (smoking cessation advice/counselling + cardiac rehabilitation + diet)	Patients who receive counseling regarding smoking cessation, referral to a cardiac rehabilitation program, and are advised on appropriate diet for secondary prevention prior to discharge.	4-5			
**Post-AMI Care**					
**Quality Indicators**				**Definition**	**References**
1. At least 1 visit to healthcare provider				Patients who receive at least one follow-up by a physician within one year from hospitalization.	4-5
2. Echocardiogram performed after discharge to reassess ventricular function				Patients who receive left ventricular ejection fraction assessment by echocardiography within one year following hospitalization.	4-5
3. Long-term statins				Patients who remain on statin therapy at one year study follow-up.	4-5
4. Dual antiplatelet therapy at 12 months				Patients who remain on dual antiplatelet therapy at one year study follow-up.	4-5

Legend: Specifically, the quality indicators of care available in the merged harmonized dataset included for the in-hospital phase: i) time to primary percutaneous coronary intervention (PCI) within 90 minutes or less for ST-segment elevation myocardial infarction (STEMI) patients, time to fibrinolytic therapy within

© 2021 Raparelli V et al. *JAMA Network Open*.

30 minutes or less for STEMI patients, angiographic assessment for NSTEMI patients, stress test in conservatively treated patients regardless the type of AMI, evaluation of left ventricular systolic function through echocardiogram, pharmacological therapy at discharge (i.e. aspirin, P2Y12 receptor inhibitor, β-blocker, statin), guideline-based recommended counselling at discharge (including adult smoking cessation advice, counseling diet and cardiac rehabilitation referral). The quality indicators evaluated for the post-AMI phase were instead at least one visit to a healthcare provider within 12-months, long term statins use, dual antiplatelet therapy at 12-months, and an echocardiogram performed within 12 months from discharge to reassess left ventricular function (post-AMI phase).

© 2021 Raparelli V et al. *JAMA Network Open*.

eTable 2. Univariate Association Among Sex, Social Determinants of Health, and the Lowest In-Hospital QCS Tertile

**Variables**	**OR**	**95% CI**		**P-value**
Age (years)	0.993	0.981	1.005	0.25
GRACE Score	1.012	1.008	1.016	<.001
Social Support (ESSI)	0.998	0.986	1.010	0.76
BMI (kg/m^2)	1.014	1.004	1.024	0.004
Support for household chores	1.141	0.977	1.333	0.09
US (vs Canada)	3.871	3.090	4.850	<.001
Self-reported White Race (vs non-White)	0.722	0.610	0.855	<.001
Low SES	1.381	1.187	1.607	<.001
Low Emotional Support	0.963	0.802	1.155	0.68
Sex male	0.554	0.475	0.645	<.001
Prior MI	1.421	1.191	1.696	<.001
Family History of CVD	1.286	1.105	1.496	0.001
Diabetes	1.421	1.218	1.657	<.001
Hypertension	1.572	1.348	1.834	<.001
Smoking	1.157	0.992	1.350	0.06
Obesity	1.132	0.978	1.309	0.09
Dyslipidemia	1.300	1.080	1.564	0.006
History of Depression	1.433	1.235	1.663	<.001
NSTEMI (vs STEMI)	6.823	5.732	8.123	<.001
High Burden of Stress	1.032	0.892	1.195	0.67
Married or Living with Partner	0.911	0.787	1.055	0.21
History of Renal Disease	1.541	1.222	1.942	<.001
Current Symptoms of Depression	1.069	0.882	1.295	0.49
Alcohol Abuse	0.882	0.756	1.028	0.11
Physically Active	0.710	0.609	0.827	<.0001
Employment	0.592	0.510	0.687	<.0001
Primary Earner Status	1.032	0.875	1.218	0.70

Abbreviations: GRACE: Global Registry of Acute Coronary Events. ESSI: ENRICHD Social Support Instrument. BMI: Body Mass Index. US: United States. MI: Myocardial Infarction. STEMI: ST Elevation Myocardial Infarction. NSTEMI: Non-ST Elevation Myocardial Infarction; CVD: Cardiovascular Disease

© 2021 Raparelli V et al. *JAMA Network Open*.

eTable 3. Multivariable Model with Imputed Values for the Lowest In-Hospital QCS Tertile

Variables	OR	95% CI		P-Value
Age (years)	0.992	0.979	1.006	0.26
BMI (kg/m^2)	0.999	0.988	1.010	0.84
US (vs Canada)	2.580	2.002	3.324	<.001
Self-reported White Race (vs non-White)	0.939	0.776	1.136	0.52
Low SES	0.840	0.700	1.008	0.06
Female Sex	1.063	0.894	1.264	0.48
Prior MI	1.099	0.888	1.360	0.39
Family History of CVD	0.950	0.793	1.139	0.58
Diabetes	0.994	0.803	1.231	0.96
Hypertension	1.194	0.995	1.433	0.06
Smoking	1.197	1.001	1.431	0.05
Dyslipidemia	0.746	0.601	0.925	0.008
History of Depression	1.101	0.925	1.310	0.28
STEMI (vs NSTEMI)	0.192	0.157	0.235	<.001
History of Renal Disease	1.199	0.895	1.605	0.23
Being Physically Active	0.930	0.765	1.130	0.47
Currently Employed	0.723	0.595	0.879	0.001

Abbreviations: QCS: Quality of Care Score; BMI: Body Mass Index. US: United States. SES: Socioeconomic Status. MI: Myocardial Infarction. CVD: Cardiovascular Disease. STEMI: ST Elevation Myocardial Infarction. NSTEMI: Non-ST Elevation Myocardial Infarction;

© 2021 Raparelli V et al. *JAMA Network Open*.

eTable 4. Multivariable Model for the Lowest In-Hospital QCS Tertile Using Random Effect for Within-Hospital Clustering.

Variables	OR	95% CI		P-Value
Age (years)	0.985	0.969	1	0.05
BMI (kg/m^2)	1.006	0.993	1.019	0.39
US (vs Canada)	2.383	1.321	4.298	0.004
Self-reported White Race (vs non-White)	1.004	0.788	1.279	0.97
Low SES	0.842	0.677	1.049	0.12
Female Sex	1.141	0.926	1.405	0.22
Prior MI	1.087	0.859	1.375	0.48
Family History of CVD	0.91	0.746	1.11	0.35
Diabetes	0.94	0.762	1.16	0.56
Hypertension	1.093	0.88	1.356	0.42
Smoking	1.202	0.979	1.476	0.08
Dyslipidemia	0.868	0.666	1.131	0.29
History of Depression	1.125	0.922	1.372	0.24
STEMI (vs NSTEMI)	0.128	0.103	0.158	<.001
History of Renal Disease	1.149	0.852	1.551	0.36
Being Physically Active	1.044	0.85	1.282	0.68
Currently Employed	0.72	0.579	0.895	0.003

Abbreviations: QCS: Quality of Care Score; BMI: Body Mass Index. SES: Socioeconomic Status. MI: Myocardial Infarction. CVD: Cardiovascular Disease. STEMI: ST Elevation Myocardial Infarction. NSTEMI: Non-ST Elevation Myocardial Infarction.

© 2021 Raparelli V et al. *JAMA Network Open*.

eTable 5. Two-Way Country-by-Sex and Individual SDOH-by-Country Interactions for the Lowest In-Hospital QCS Tertile

	**Lower In-Hospital QCS P-value Interaction**
**Country*Sex**	0.21
**Country*Individual SDOH**	
▪ Low SES	0.66
▪ Current employment	0.73
▪ Number of work hrs/wk	0.26
▪ Married or living with Partner	0.27
▪ Primary earner	0.65
▪ High burden of stress	0.46
▪ Support for household chores	0.58
▪ Low social support	0.50

Abbreviations: QCS: Quality of Care Score. SDOH: Social Determinants of Health. SES: Socio-economic Status.

© 2021 Raparelli V et al. *JAMA Network Open*.

eTable 6. Univariate Association Among Sex, Social Determinants of Health, and the Lowest Post-AMI QCS Tertile

Variables	OR	95% CI		P-Value
Age (years)	0.991	0.978	1.005	0.21
GRACE Score	1.006	1.002	1.011	0.005
Social Support (ESSI)	0.981	0.967	0.995	0.007
BMI (kg/m^2)	1.008	0.997	1.019	0.15
Support for household chores	0.825	0.693	0.981	0.03
US (vs Canada)	3.031	2.325	3.950	<.001
White Race (vs nonn-White)	0.682	0.560	0.829	<.001
Low SES	1.521	1.279	1.808	<.001
Low Emotional Support	0.898	0.730	1.103	0.30
Sex male	0.707	0.594	0.842	<.001
Prior MI	1.488	1.214	1.825	<.001
Family History of CVD	1.127	0.949	1.340	0.17
Diabetes	1.284	1.075	1.533	0.006
Hypertension	1.213	1.021	1.441	0.03
Smoking	1.116	0.937	1.331	0.22
Obesity	1.035	0.876	1.222	0.69
Dyslipidemia	1.124	0.913	1.383	0.27
History of Depression	1.233	1.040	1.463	0.016
NSTEMI (vs STEMI)	1.572	1.330	1.858	<.001
High Burden of Stress	1.093	0.924	1.293	0.30
Married or living with Partner	0.661	0.559	0.781	<.001
History of Renal Disease	1.251	0.954	1.640	0.10
Current symptoms of depression	1.082	0.868	1.350	0.48
Alcohol Abuse	0.920	0.772	1.096	0.35
Physically Active	0.738	0.618	0.881	<.001
Employment	0.750	0.630	0.892	<.001
Primary Earner Status	0.844	0.700	1.017	0.07

Abbreviations: GRACE: Global Registry of Acute Coronary Events. ESSI: ENRICHD Social Support Instrument. BMI: Body Mass Index. US: United States. SES: Socio-economic Status. MI: Myocardial Infarction. STEMI: ST Elevation Myocardial Infarction. NSTEMI: Non-ST Elevation Myocardial Infarction; CVD: Cardiovascular Disease;

© 2021 Raparelli V et al. *JAMA Network Open*.

eTable 7. Multivariable Model With Imputed Values for the Lowest Post-AMI QCS Tertile

	**OR**	**95%CI**		**P-value**
Age (years)	0.997	0.982	1.012	0.71
Social Support (ESSI)	0.988	0.973	1.004	0.14
US (vs Canada)	2.672	1.967	3.629	<.001
Self-reported White Race (vs non-White)	0.756	0.608	0.939	0.012
Low SES	1.134	0.920	1.399	0.24
Female Sex	1.066	0.875	1.299	0.53
Prior MI	1.283	1.023	1.609	0.031
Diabetes	0.990	0.812	1.208	0.92
Hypertension	0.926	0.761	1.128	0.45
Depression	1.016	0.837	1.233	0.87
STEMI (vs NSTEMI)	0.688	0.576	0.823	<.001
Being Physically Active	0.896	0.739	1.087	0.26
Currently Employed	0.997	0.809	1.229	0.98

Abbreviations: ESSI: ENRICHD Social Support Instrument. US: United States. SES: Socioeconomic Status. MI: Myocardial Infarction. CVD: Cardiovascular Disease. STEMI: ST Elevation Myocardial Infarction. NSTEMI: Non-ST Elevation Myocardial Infarction;

© 2021 Raparelli V et al. *JAMA Network Open*.

eTable 8. Multivariable Model for the Lowest Post-AMI QCS Tertile Using Random Effect for Within-Hospital Clustering

	**OR**	**95%CI**		**P-value**
Age (years)	0.997	0.982	1.012	0.68
Social Support (ESSI)	0.988	0.972	1.004	0.13
US (vs Canada)	2.684	1.856	3.880	<.001
Self-reported White Race (vs non-White)	0.756	0.602	0.950	0.016
Low SES	1.134	0.916	1.406	0.25
Female Sex	1.073	0.878	1.312	0.49
Prior MI	1.284	1.020	1.618	0.034
Diabetes	0.985	0.804	1.205	0.88
Hypertension	0.920	0.752	1.125	0.42
Depression	1.021	0.838	1.244	0.84
STEMI (vs NSTEMI)	0.691	0.576	0.830	<.001
Being Physically Active	0.901	0.739	1.099	0.30
Currently Employed	0.995	0.803	1.232	0.96

Abbreviations: ESSI: ENRICHD Social Support Instrument. US: United States. SES: Socioeconomic Status. MI: Myocardial Infarction. CVD: Cardiovascular Disease. STEMI: ST Elevation Myocardial Infarction. NSTEMI: Non-ST Elevation Myocardial Infarction.

© 2021 Raparelli V et al. *JAMA Network Open*.

eTable 9. Two-Way Country-by-Sex and Individual SDOH-by-Country Interactions for the Lowest Post-AMI QCS Tertile

	**Lower Post-AMI QCS P-value Interaction**
**Country*Sex**	0.08
**Country*Individual SDOH**	
▪ Low SES	0.70
▪ Current employment	0.68
▪ Number of work hrs/wk	0.58
▪ Married or living with Partner	0.89
▪ Primary earner	0.52
▪ High burden of stress	0.93
▪ Support for household chores	0.88
▪ Low social support	0.62

Abbreviations: QCS: Quality of Care Score. SDOH: Social Determinants of Health. SES: Socio-economic Status.

© 2021 Raparelli V et al. *JAMA Network Open*.

eTable 10. Data Dictionary and Summary of the Harmonized Variables

Variable Label	NEW Variable PRAXY-VIRGO Merged Data	Definition	Type of variable
Country Single-payer vs Multi-payer	PVM_InsurancePublicPrivate	1=public 2=private	categorical
Type of Myocardial Infarction	PVM_TypeMI	1= STEMI<br>2= NSTEMI	categorical
Education Level 3 categories	PVM_LevelEducation	No High school (low education)= 0<br>High school (intermediate education) = 1<br>More than High school (high education) = 2	categorical
Income	PVM_Income	Low (≤30,000 USD or ≤30,000 CAD)= 1<br>Intermediate-High (>30,000 USD or >30,000 CAD) = 2	categorical
Low SES (Combining Low education and Low income)	PVM_LowSES	yes (low education and/or Low income)=1<br>no (high education and/or high income)=0	dichotomic
Sex	PVM_Sex	Male = 1<br>Female = 0	categorical
Age (years)	PVM_Age	years at enrollment	numeric
Ethnicity/Race	PVM_EthnicityRace	Black / African American =1<br>White / Caucasian = 2<br>American Indian / Alaska Native = 3<br>Asian / Pacific Islander / East Indian = 4<br>Other = 5<br>Don't Know = 6	categorical
Height	PVM_Height	Height cm (baseline)	numeric
Weight	PVM_Weight	Weight kg	numeric
BMI (kg/m^2)	PVM_BMI	BMI kg/m^2	numeric
Obesity	PVM_Obese	BMI ≥ 30 = 1, BMI < 30 = 0	dichotomic
Hypertension	PVM_Hypertension	No=0<br>Yes=1	dichotomic
Diabetes	PVM_Diabetes	No=0<br>Yes=1	dichotomic
Dyslipidemia	PVM_Dyslipidemia	No=0<br>Yes=1	dichotomic
Previous MI	PVM_PreviousMI	No=0<br>Yes=1	dichotomic
Smoking	PVM_Smoking	No=0<br>Yes=1	dichotomic
Alcohol consumption more than 2 drinks per day	PVM_Alcohol2perDay	No=0<br>Yes=1	dichotomic
Family History of CVD	PVM_FamHistCVD	No=0<br>Yes=1	dichotomic
Depression	PVM_Depression	No=0<br>Yes=1	dichotomic
Physically Active vs Inactive	PVM_PhysicallyActive	No=0<br>Yes=1	dichotomic
History of Renal Disease	PVM_HistRenalDisease	No=0<br>Yes=1	dichotomic

© 2021 Raparelli V et al. *JAMA Network Open*.

Married or living in common law	PVM_MaritalStatus	No=0<br>Yes=1	dichotomic
Working	PVM_WorkingStatus	No=0<br>Yes=1	dichotomic
Household Primary earner	PVM_PrimaryEarner	No=0<br>Yes=1	dichotomic
Hours Per Week at Work	PVM_WorkHoursWeek	hours	number
Level of stress overall	PVM_StressOverall	No=0<br>Yes=1	dichotomic
Depression Symptoms - Feeling Down	PVM_FeelDown	No=0<br>Yes=1	dichotomic
Depression Symptoms - Lost Interest	PVM_LoseInterest	No=0<br>Yes=1	dichotomic
Depression Symptoms - Trouble Sleeping	PVM_Sleep	No=0<br>Yes=1	dichotomic
Depression Symptoms - Trouble Concentrating	PVM_Concentrate	No=0<br>Yes=1	dichotomic
Depression scale 2WEEKS; change Weight	PVM_WeightApetit	No=0<br>Yes=1	dichotomic
Depression Symptoms - Feeling Worthless	PVM_Worthless	No=0<br>Yes=1	dichotomic
Depression Symptoms - Feeling Tired	PVM_Tired	No=0<br>Yes=1	dichotomic
Depression Symptoms - Think Death	PVM_ThinkDeath	No=0<br>Yes=1	dichotomic
DSM-IV Major Depression	PVM_MajorDepression	No=0<br>Yes=1	dichotomic
ESSI Someone available to listen to you	PVM_Listen	None of the time =1<br>A little of the time =2<br>Some of the time =3<br>Most of the time= 4<br>All of the time =5	categorical
ESSI Someone available to give you advice	PVM_Advice	None of the time =1<br>A little of the time =2<br>Some of the time =3<br>Most of the time= 4<br>All of the time =5	categorical
ESSI Someone available to give you affection	PVM_Affection	None of the time =1<br>A little of the time =2<br>Some of the time =3<br>Most of the time= 4<br>All of the time =5	categorical
ESSI Someone available that you can trust and confide in	PVM_TrustConfide	None of the time =1<br>A little of the time =2<br>Some of the time =3<br>Most of the time= 4<br>All of the time =5	categorical
ESSI Level of emotional support	PVM_EmotionSupport	None of the time =1<br>A little of the time =2<br>Some of the time =3<br>Most of the time= 4<br>All of the time =5	categorical
Total score ESSI 5 items	PVM_ESSI_5_Total	scale between 5 and 25	numeric
Low Social Support (5 items)	PVM_LowSocialSupport	No=0<br>Yes=1	dichotomic

© 2021 Raparelli V et al. *JAMA Network Open*.

ESSI 7 Someone available to you to help you with daily chores	PVM_Chores	None of the time =1<br>A little of the time =2<br>Some of the time =3<br>Most of the time= 4<br>All of the time =5	categorical
ESSI 7 Married	PVM_ESSIMarried	yes = 1<br>no =0	categorical
ESSI 7 Total score	PVM_ESSI_7_Total	None of the time =1<br>A little of the time =2<br>Some of the time =3<br>Most of the time= 4<br>All of the time =5	categorical
Thrombolysis in STEMI	PVM_ThrombolysisSTEMI	Reperfusion Therapy with thrombolytics in STEMI = 1	categorical
Any reperfusion strategy in STEMI	PVM_ReperfusionSTEMI	if PVM_ThrombolysisSTEMI =1 or if PVM_PPCI =1 then PVM_ReperfusionSTEMI =1	categorical
Reperfusion among those with STEMI (primary PCI)	PVM_PPCI	No=0<br>Yes=1	dichotomic
Door to needle, in minutes	PVM_Door2Needlemin	number in minutes	numeric
Exceed Door to Needle requirements	PVM_Door2NeedleExceed	No=0<br>Yes=1	dichotomic
Door to needle, in Minutes, STEMI only	PVM_Door2NeedleminSTEMI	number of minutes	numeric
Exceed Door to Needle requirements, STEMI only	PVM_Door2NeedleExceedSTEMI	No=0<br>Yes=1	dichotomic
Door to balloon, in minutes, STEMI only	PVM_Door2BaloonminSTEMI	number of minutes	numeric
Exceed Door to Balloon requirements, STEMI only	PVM_Door2BaloonExceedSTEMI	No=0<br>Yes=1	dichotomic
GRACE score in NSTEMI performed	PVM_GRACENSTEMI	No=0<br>Yes=1	dichotomic
PCI among NSTEMI	PVM_PCINSTEMI	No=0<br>Yes=1	dichotomic
GRACE score > 140 in NSTEMI	PVM_GRACENSTEMI140HIghRisk	No=0<br>Yes=1	dichotomic
Echo Performed Pre-Discharge	PVM_EchoPreDischarge	No=0<br>Yes=1	dichotomic
Stress Test in Conservatively Treated Patients	PVM_ConservativeStressTest	No=0<br>Yes=1	dichotomic
Medication Counseling at Discharge	PVM_CounselingMeds	No=0<br>Yes=1	dichotomic
Dietary Counseling at Discharge	PVM_DietCounseling	No=0<br>Yes=1	dichotomic
Exercise Counseling at Discharge	PVM_Activity guidelines	No=0<br>Yes=1	dichotomic
Cardiac Rehab. Counseling	PVM_OutPatientCardiacRehab	No=0<br>Yes=1	dichotomic
Smoking Cessation Counseling at Discharge	PVM_SmokingCessationCounseling	No=0<br>Yes=1	dichotomic
Recommended counseling (including cardiac rehab, smoking cession, diet)	PVM_RecommendedCounseling	No=0<br>Yes=1	dichotomic
ASA at Discharge	PVM_MedDischargeASA	No=0<br>Yes=1	dichotomic
Clopidogrel at Discharge	PVM_MedDischargeCLOP	No=0<br>Yes=1	dichotomic

© 2021 Raparelli V et al. *JAMA Network Open*.

Statins at discharge	PVM_MedDischargeStatins	No=0<br>Yes=1	dichotomic
Beta-blockers at Discharge	PVM_MedDischargeBetaBlockers	No=0<br>Yes=1	dichotomic
Echo Post AMI	PVM_PostACSDoneEcho	No=0<br>Yes=1	dichotomic
Number of echo Post AMI discharge	PVM_PostACSNumberEcho	n. echo performed	numeric
Family Physician Post AMI	PVM_PostACSFamMDVisits	n. visits	numeric
Cardiovascular specialists (e.g. Cardiologist, Cardiac surgeon) Post AMI	PVM_PostACSCardioSpecialVisits	n. visits	numeric
ASA prescribed at 12months	PVM_Med12monthASA	No=0<br>Yes=1	dichotomic
Clopidogrel prescribed at 12months	PVM_Med12monthCLOP	No=0<br>Yes=1	dichotomic
Statins prescribed at 12months	PVM_Med12monthStatins	No=0<br>Yes=1	dichotomic

© 2021 Raparelli V et al. *JAMA Network Open*.

eReferences

1. Wadhera RK, Bhatt DL, Wang TY, et al. Association of State Medicaid Expansion With Quality of Care and Outcomes for Low-Income Patients Hospitalized With Acute Myocardial Infarction. JAMA Cardiology. 2019; 4(2):120-127.

2. Jneid H, Addison D, Bhatt DL, et al. 2017 AHA/ACC Clinical Performance and Quality Measures for Adults With ST-Elevation and Non-ST-Elevation Myocardial Infarction: A Report of the American College of Cardiology/American Heart Association Task Force on Performance Measures. J Am Coll Cardiol. 2017;70(16):2048-2090

3. Schiele F, Gale CP, Bonnefoy E, et al. Quality indicators for acute myocardial infarction: A position paper of the Acute Cardiovascular Care Association. Eur Heart J Acute Cardiovasc Care. 2017;6(1):34-59

4. Antman EM, Anbe DT, Armstrong PW, et al. ACC/AHA guidelines for the management of patients with ST-elevation myocardial infarction--executive summary: a report of the American College of Cardiology/American Heart Association Task Force on Practice Guidelines (Writing Committee to Revise the 1999 Guidelines for the Management of Patients With Acute Myocardial Infarction). Circulation. 2004;110(5):588-636.

5. Anderson JL, Adams CD, Antman EM, et al. ACC/AHA 2007 guidelines for the management of patients with unstable angina/non ST-elevation myocardial infarction: a report of the American College of Cardiology/American Heart Association Task Force on Practice Guidelines (Writing Committee to Revise the 2002 Guidelines for the Management of Patients With Unstable Angina/Non ST-Elevation Myocardial Infarction): developed in collaboration with the American College of Emergency Physicians, the Society for Cardiovascular Angiography and Interventions, and the Society of Thoracic Surgeons: endorsed by the American Association of Cardiovascular and Pulmonary Rehabilitation and the Society for Academic Emergency Medicine. Circulation. 2007;116(7):e148-304.

© 2021 Raparelli V et al. *JAMA Network Open*.
